# Incorporating tumour pathology information into breast cancer risk prediction algorithms

**DOI:** 10.1186/bcr2576

**Published:** 2010-05-18

**Authors:** Nasim Mavaddat, Timothy R Rebbeck, Sunil R Lakhani, Douglas F Easton, Antonis C Antoniou

**Affiliations:** 1 Centre for Cancer Genetic Epidemiology, Department of Public Health and Primary Care, University of Cambridge, Strangeways Research Laboratory, Worts Causeway, Cambridge CB1 8RN, UK; 2 Center for Clinical Epidemiology and Biostatistics, Department of Biostatistics and Epidemiology, University of Pennsylvania, 217 Blockley Hall, 423 Guardian Drive, Philadelphia, PA 19104, USA; 3 Molecular and Cellular Pathology, University of Queensland Centre for Clinical Research, Level 6 Building 71/918, University of Queensland, The Royal Brisbane & Women's Hospital, Herston, 4029 Brisbane, Queensland, Australia

## Abstract

**Introduction:**

Mutations in *BRCA1 *and *BRCA2 *confer high risks of breast cancer and ovarian cancer. The risk prediction algorithm BOADICEA (Breast and Ovarian Analysis of Disease Incidence and Carrier Estimation Algorithm) may be used to compute the probabilities of carrying mutations in *BRCA1 *and *BRCA2 *and help to target mutation screening. Tumours from *BRCA1 *and *BRCA2 *mutation carriers display distinctive pathological features that could be used to better discriminate between *BRCA1 *mutation carriers, *BRCA2 *mutation carriers and noncarriers. In particular, oestrogen receptor (ER)-negative status, triple-negative (TN) status, and expression of basal markers are predictive of *BRCA1 *mutation carrier status.

**Methods:**

We extended BOADICEA by treating breast cancer subtypes as distinct disease end points. Age-specific expression of phenotypic markers in a series of tumours from 182 *BRCA1 *mutation carriers, 62 *BRCA2 *mutation carriers and 109 controls from the Breast Cancer Linkage Consortium, and over 300,000 tumours from the general population obtained from the Surveillance Epidemiology, and End Results database, were used to calculate age-specific and genotype-specific incidences of each disease end point. The probability that an individual carries a *BRCA1 *or *BRCA2 *mutation given their family history and tumour marker status of family members was computed in sample pedigrees.

**Results:**

The cumulative risk of ER-negative breast cancer by age 70 for *BRCA1 *mutation carriers was estimated to be 55% and the risk of ER-positive disease was 18%. The corresponding risks for *BRCA2 *mutation carriers were 21% and 44% for ER-negative and ER-positive disease, respectively. The predicted *BRCA1 *carrier probabilities among ER-positive breast cancer cases were less than 1% at all ages. For women diagnosed with breast cancer below age 50 years, these probabilities rose to more than 5% in ER-negative breast cancer, 7% in TN disease and 24% in TN breast cancer expressing both CK5/6 and CK14 cytokeratins. Large differences in mutation probabilities were observed by combining ER status and other informative markers with family history.

**Conclusions:**

This approach combines both full pedigree and tumour subtype data to predict *BRCA1/*2 carrier probabilities. Prediction of *BRCA1/2 *carrier status, and hence selection of women for mutation screening, may be substantially improved by combining tumour pathology with family history of cancer.

## Introduction

Genetic testing for *BRCA1 *and *BRCA2 *has important clinical implications: individuals found to carry mutations in these genes can be carefully monitored and receive preventive therapies including oophorectomy or mastectomy [1-6]. As genetic testing is expensive and may be associated with adverse psychological effects for the individual and their family, testing is only appropriate for those at highest risk of carrying mutations.

Several models have been developed for predicting risk of carrying *BRCA1 *or *BRCA2 *mutation and subsequently developing breast cancer [7]. These models generally include information on occurrence and age of diagnosis of breast cancer and other cancers in individuals and their families [7]. Breast tumours arising in carriers of *BRCA1 *or *BRCA2 *mutations, however, also differ from one another and from sporadic tumours in terms of their pathological characteristics, including those assessed morphologically or by immunohistochemistry [8-12]. Incorporating information about the pathology of breast tumours in the proband or family members into risk-prediction algorithms may result in improved discrimination between *BRCA1 *mutation carriers, *BRCA2 *mutation carriers and nonmutation carriers, and provide a more accurate basis for identifying those individuals that may benefit from genetic testing.

Numerous studies have linked the absence of oestrogen receptor (ER) expression in breast tumours with *BRCA1 *mutation carrier status using different laboratory methods, anti-ER antibodies, and cut-off points for ER staining [13-22]. In the largest study to date, the Breast Cancer Linkage Consortium (BCLC) reported that ER-negativity - defined by <1% of breast cancer cells expressing detectable ER by immunohistochemistry - conferred an age-adjusted odds ratio of 13.98 (95% confidence interval, 7.1 to 27.7; *P *< 0.0001) for *BRCA1 *mutation carrier status compared with controls unselected for family history (FH) [10]. Furthermore, the ER status was found to be the most significant risk factor in multiple regression analyses including other factors that are individually significant predictors of *BRCA1 *status. These factors included progesterone receptor, human epidermal growth factor receptor 2 (HER2), mitotic count, lymphocyte infiltration, and continuous pushing margins [10].

The triple-negative (TN) tumours are negative for ER, progesterone receptor, and HER2, and they define a subset of ER-negative disease. Subsets of TN tumours also express high-molecular-weight cytokeratins (CKs) (for example, CK5/6 and CK14), also referred to as basal CKs. Expression of basal CKs in TN tumours has been shown to provide additional information predictive of *BRCA1 *status [11]. The distribution of ER status in breast tumours and CK expression amongst TN tumours from *BRCA2 *mutation carriers appears to be similar to that in tumours from the population overall [10,11]. HER2-positive tumours have been shown less common in both *BRCA1 *and *BRCA2 *mutation carriers than in noncarriers [10].

We previously developed a risk-prediction algorithm for familial breast cancer and ovarian cancer - the Breast and Ovarian Analysis of Disease Incidence and Carrier Estimation Algorithm (BOADICEA) - which can be used to compute the probabilities of carrying *BRCA1 *and *BRCA2 *mutations and the probabilities of developing breast cancer or ovarian cancer in the future [23]. BOADICEA models the effects of *BRCA1 *and *BRCA2 *mutations as well as a polygenic component representing the joint multiplicative effect of a large number of genes each of small effect [24,25]. BOADICEA has recently been extended to incorporate the associations between *BRCA1 *and *BRCA2 *mutations and other cancer risks (such as prostate cancer, pancreatic cancer and male breast cancer) [23], and has been shown to discriminate well between mutation carriers and noncarriers in a large series of families identified through UK genetic clinics [7,26]. In the present report we propose a method for incorporating breast tumour pathology information into the BOADICEA risk-prediction model.

## Materials and methods

### Age-specific distribution of ER status and distribution of other markers

Data from the BCLC [10,11] were used to obtain the age-specific proportions of ER-negative and ER-positive tumours, and the proportions of TN tumours and CK5/6-expressing and/or CK14-expressing tumours for *BRCA1 *or *BRCA2 *carriers or controls. Breast cancer patients with *BRCA1 *and *BRCA2 *mutations were identified through families with multiple relatives diagnosed with breast cancer or ovarian cancer. Data on 182 tumours in *BRCA1 *mutation carriers from 119 families, 62 tumours from *BRCA2 *mutation carriers from 35 families, and 109 control women with breast cancer who were unselected for FH and not tested for mutation carrier status were available.

Details of the methods used to determine tumour marker expression are described by Lakhani and colleagues [10,11]. Briefly, assays were performed at a single, quality-assured laboratory, and both the proportion of tumour cells staining with ER, progesterone receptor, HER2, CK5/6 or CK14 antibodies and the intensity of staining as assayed by immunohistochemistry were recorded [10,11]. The present work had been carried out under local ethical approval. Owing to the sparse data on *BRCA2 *tumours, and since the proportion of ER-positive tumours were not significantly different from that in controls [10], we assumed in the model that the age-specific proportions of ER-positive tumours and ER-negative tumours were the same as the proportions in the general population.

Age-specific proportions of ER-negative cancers and ER-positive cancers in the general population were obtained from the Surveillance Epidemiology, and End Results (SEER) database, a large population-based US breast cancer registry [27]. Data for the years 1990 to 2006 (n = 326,839) were used. Data were coded as ER-positive, ER-negative, or borderline. All analyses were restricted to females of white ethnicity with invasive breast cancer.

### Statistical methods

#### Extending BOADICEA to incorporate tumour pathology

BOADICEA models the genetic susceptibility to breast cancer in terms of *BRCA1*, *BRCA2 *and a polygenic component representing the combined multiplicative effect of several loci, each of small effect [23].

The breast cancer incidence for individual *i *is assumed to depend on the underlying genotype through a model of the form:

where *λ*_0_(*t*) is the baseline incidence at age *t*, *G*_*i*_(*t*) is the log relative risk corresponding to the major genotype (that is, *BRCA1 *carrier, *BRCA2 *carrier or nonmutation carrier) at age *t*, and *P*_*i*_(*t*) is the age-dependent polygenic effect assumed to be normally distributed with mean zero and variance *σ*^2^(*t*). The polygenic component is approximated by the hypergeometric polygenic model [28].

The incidence of ovarian cancer is modelled in a similar manner, but without a polygenic component.

The probabilities of developing breast cancer and ovarian cancer are assumed to be independent, conditional on the underlying genetic effects. Cancer incidences in BOADICEA are calendar and cohort specific. The overall age-specific incidences, averaged over all major genotypes and polygenotypes, are constrained to agree with the population incidences for England and Wales [29-35]. The BOADICEA model is implemented in the pedigree analysis program MENDEL v3.3 [36]. Additional model description and the model parameter estimates can be found in our previous publication [23].

To incorporate tumour phenotypes, we considered breast cancer subtypes as different disease end points. For example, to incorporate the ER status, the total observed breast cancer incidence was assumed to be:

where *μ*(*t*) is the incidence of ER-positive disease and *ν*(*t*) is the incidence of ER-negative disease. We also assumed that the probabilities of developing ER-negative or ER-positive breast cancer are independent, conditional on the underlying genotype. We assumed that the polygenic component *P*_*i*_(*t*) was identical for both ER-negative and ER-positive disease (that is, represented by the same set of polygenes conferring the same relative risks), although in principle this assumption could be relaxed. The incidence for each individual *i *at age *t *therefore follows a model of the form:

where *μ*_*i*_(*t*) and *ν*_*i*_(*t*) are the major genotype-specific incidences for ER-positive and ER-negative disease, respectively, for individual *i*.

Given the existing genotype-specific (*BRCA1 *carriers, *BRCA2 *carriers, and noncarriers) incidences in BOADICEA and the age-specific distribution of ER status in breast tumours from the BCLC and SEER data, our aim was to derive separate genotype-specific incidences for ER-positive disease and ER-negative disease. These were obtained by constraining the overall *BRCA1 *and *BRCA2 *incidences over ER status and polygenic effects to equal the average breast cancer incidence for *BRCA1 *and *BRCA2 *mutation carriers, estimated previously in BOADICEA; and by constraining the overall incidence over ER status, major gene (*BRCA1 *carriers, *BRCA2 *carriers and noncarriers) and polygenic effects to agree with the population breast cancer incidences. Separate genotype-specific incidences were derived for TN ER-negative disease and non-TN ER-negative disease as well as for CK5/6- and/or CK14-expressing and non-expressing TN tumours using a similar approach. Details of the method are provided in Additional file 1.

The derived incidences were used in the penetrance calculations whenever information on the relevant tumour marker status was available. For individuals with no marker status information, penetrance calculations use the total breast cancer incidences (as in the standard BOADICEA model implementation [23]). The genetic model is therefore fully specified by the *BRCA1 *and *BRCA2 *mutation frequencies, the polygenic variance and genotype-specific incidences for each type of disease.

#### BRCA1 and BRCA2 mutation carrier probabilities

The probability that an individual carries a *BRCA1 *or *BRCA2 *mutation given FH and marker status (for example, ER status), was computed as follows:

where *P*(*BRCAj*, *FH*, *ER*) is the probability of observing the family with the particular FH and ER status, and the proband carrying a *BRCA j *mutation (*BRCA j *= *BRCA1 *carrier, *BRCA2 *carrier, or noncarrier). These correspond to pedigree likelihoods generated using the MENDEL program [36].

#### Calculations for example pedigrees

We investigated the effect of incorporating tumour phenotypic information on the mutation carrier probabilities using a variety of different scenarios. Carrier probabilities were calculated in simple pedigrees for scenarios where the marker status of tumours is unknown, or is known in one or more members of a family. For simplicity, we assumed that there was no follow-up after the age of diagnosis of cancer in each case.

## Results

### ER status of tumours from mutation carriers and unselected breast cancers in the general population

Age-specific proportions of ER-negative tumours in *BRCA1 *mutation carriers and *BRCA2 *mutation carriers derived from the BCLC dataset are presented in Table 1. These proportions were obtained for ER status defined as positive where >1% of cells stained with anti-ER antibody in immunohistochemistry assays. Similar results were obtained when a combination of the proportion of cells staining and a staining intensity equivalent to an Allred score of 2 - a score shown to correspond to clinical response to Tamoxifen [37-39] - was used (data not shown). Data for age categories >70 years were sparse for *BRCA1 *carriers, and we extrapolated data from the age category 60 to 69 years for this group. Data for *BRCA2 *carriers were sparse in all age groups, resulting in jumps in proportions between adjacent intervals (Table 1). We therefore used population data [27] for *BRCA2 *carriers in our analyses, as *BRCA2 *carriers have been shown to have similar ER status distribution to unselected controls [10].

**Table 1:** Oestrogen receptor-negative tumours as a proportion of total tumours in *BRCA1 *and *BRCA2 *mutation carriers

Carrier	<30 years old	30 to 39 years old	40 to 49 years old	50 to 59 years old	60 to 69 years old	>70 years old
*BRCA1*	0.93	0.91	0.86	0.89	0.83	0.83^a^
*BRCA2*^a^	0.44	0.26	0.47	0.14	0.20	0.15

Data from the Breast Cancer Linkage Consortium (BCLC) [10]. ^a^As numbers were sparse, data were extrapolated from the nearest age group for *BRCA1 *carriers in updating the Breast and Ovarian Analysis of Disease Incidence and Carrier Estimation Algorithm, and population data were substituted for BCLC data for *BRCA2 *carriers.

**Figure 1:**
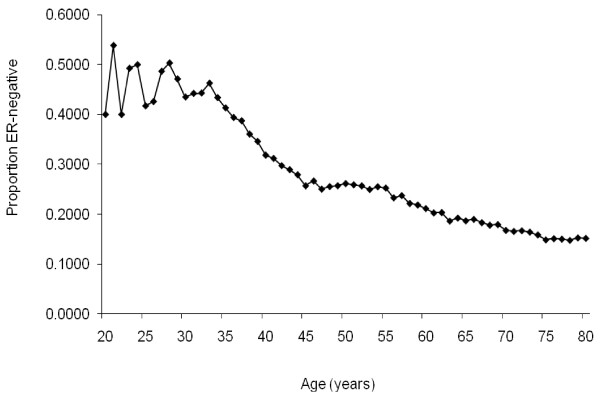
**Proportion of oestrogen receptor-negative tumours from the general population**. Oestrogen receptor (ER)-negative tumours as a proportion of all invasive breast cancers tested for ER expression in unselected females from the general population. Data from the Surveillance Epidemiology, and End Results database 1990 to 2006 [27].

The age-specific proportions of ER-negative invasive breast cancers from white females in the general population obtained from the SEER database are shown in Figure 1. As previously reported, the proportion of ER-negative tumours decreases with age [40].

### Expression of other markers in tumours from mutation carriers and unselected controls

The proportions of TN tumours amongst ER-negative tumours and of CK-expressing tumours among TN tumours derived from the BCLC dataset are presented in Table 2. These proportions were obtained for progesterone receptor, CK5/6 or CK14 - defined as positive when >1% of cells stained with the specific antibodies in immunohistochemistry - and for HER2, where the majority of cells showed a strong complete membrane staining (equivalent to score 3 on the DAKO scoring system) [10,11]. Owing to sparse data, we used a constant proportion for expression of these tumour markers across all ages at diagnosis of breast cancer. As CK expression among TN tumours in *BRCA2 *carriers was similar to the distribution among TN tumours in breast cases unselected for FH and not tested for mutation (*P *> 0.05), we used data from these controls for *BRCA2 *carriers in our analyses.

**Table 2:** Proportion of each tumour subtype in *BRCA1 *and *BRCA2 *mutation carriers and unselected controls

Tumour phenotype	Controls^a^	*BRCA1 *carriers	*BRCA2 *carriers
TN (among ER-negative cases)	0.53	0.90	0.86
CK5/6-expressing and CK14-expressing TN^b, c^	0.14	0.49	0.38^d^
Either CK5/6-expressing or CK14-expressing TN^b, c^	0.24	0.30	0.13^d^
CK5/6-expressing TN^b ^(only CK5/6 tested)	0.26	0.64	0.38^d^
CK14-expressing TN^b ^(only CK14 tested)	0.27	0.63	0.50

Data from Breast Cancer Linkage Consortium [11]. CK, cytokeratin; ER, oestrogen receptor; TN, triple negative. ^a^Controls were breast cancer cases unselected for family history and untested for mutation. ^b^As a proportion of all TN tumours. ^c^Both the CK5/6 status and the CK14 status have been tested. ^d^As numbers were sparse, proportions from controls were used in updating the Breast and Ovarian Analysis of Disease Incidence and Carrier Estimation Algorithm.

### Breast cancer subtype-specific risks

**Figure 2:**
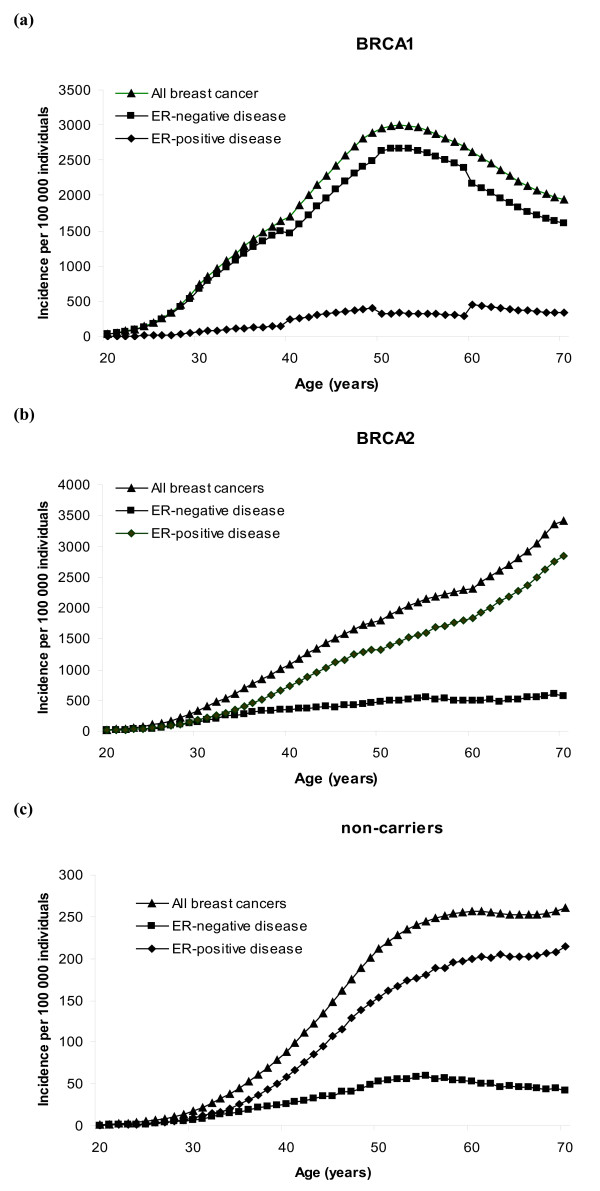
**Incidence rates of oestrogen receptor-negative disease, oestrogen receptor-positive disease and all breast cancers**. Incidence rates per 100,000 individuals of oestrogen receptor (ER)-negative disease, ER-positive disease and all breast cancers in **(a) ***BRCA1 *carriers, **(b) ***BRCA2 *carriers and **(c) **noncarriers, corresponding to women born after 1950.

ER-specific incidences were calculated for each of the five birth cohorts considered in BOADICEA. The resulting incidences (averaged over all polygenotypes) of ER-positive and ER-negative breast cancers in *BRCA1 *carriers, *BRCA2 *carriers and noncarriers for women born after 1950 are shown in Figure 2. The breast cancer incidence curve in *BRCA1 *mutation carriers follows closely the shape of the incidence curve for ER-negative disease (Figure 2a). This increases rapidly with age until about age 50 years but decreases gradually after this age. This pattern is similar to the incidence of ER-negative disease in noncarriers (Figure 2c). In contrast, the shape of the overall breast cancer incidence curve in *BRCA2 *mutation carriers is similar to that for ER-positive disease, where incidence increases with age, with an inflexion at about age 50 years (Figures 2b). This is also consistent with the age-specific pattern of ER-positive disease in noncarriers (Figure 2c) and in unselected controls [40].

**Figure 3:**
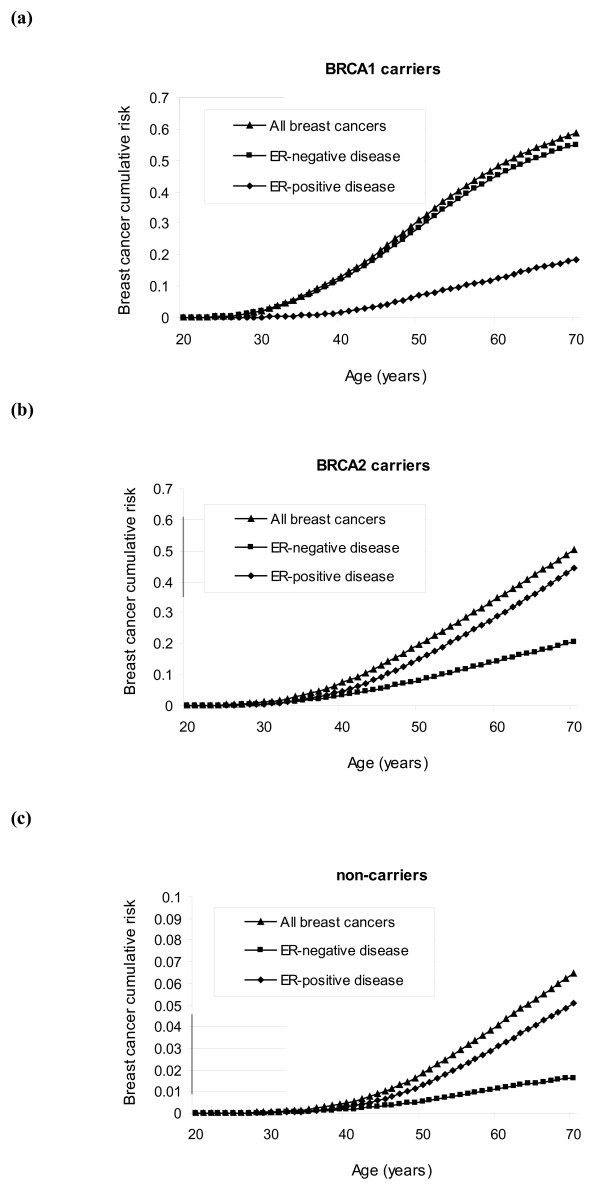
**Average cumulative risk for oestrogen receptor-negative disease, oestrogen receptor-positive disease and all breast cancers**. Average cumulative risk for oestrogen receptor (ER)-negative disease, ER-positive disease and all breast cancers in **(a) ***BRCA1 *carriers, **(b) ***BRCA2 *carriers, and **(c) **noncarriers, corresponding to women born after 1950.

Figure 3 shows the corresponding average cumulative risks of developing ER-positive and ER-negative breast cancer by genotype. The probabilities of developing ER-positive and ER-negative breast cancer for *BRCA1 *mutation carriers by age 70 were calculated to be 18% and 55%, respectively. The corresponding risks for *BRCA2 *mutation carriers were 44% and 21% for ER-positive disease and ER-negative disease, respectively. Subtype-specific incidences for TN tumours and basal CK-expressing or non-expressing TN tumours, and the corresponding average cumulative risks of developing these tumours, are provided in Additional file 2 (Supplementary figures S1 to S6).

### Prediction of BRCA1 and BRCA2 carrier probabilities

**Figure 4:**
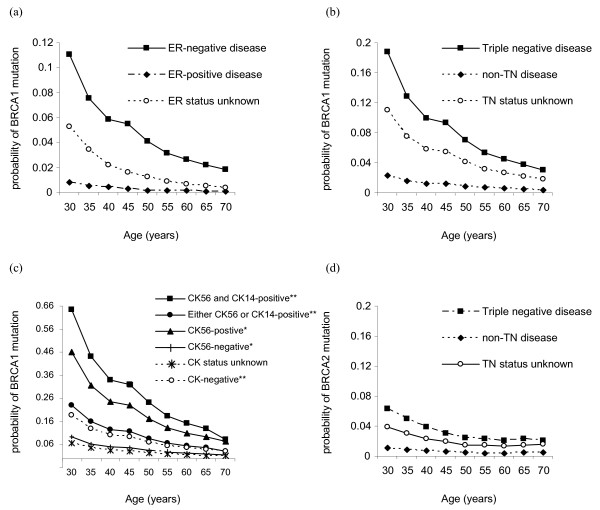
**Influence of tumour pathology on mutation carrier probabilities for a single affected individual**. Mutation carrier probabilities for a single affected individual with no knowledge of family history of breast cancer: *BRCA1 *carrier probabilities are shown in relation to **(a) **oestrogen receptor (ER) status, **(b) **tumour-negative (TN) status, when the tumour is known to be ER-negative, and **(c) **cytokeratin (CK)5/6 and/or CK14 expression when the tumour is known to be TN. In (c) scenarios where both basal markers have been tested (**) or where only CK5/6 has been tested (*) are shown. *BRCA2 *carrier probabilities are shown in relation to **(d) **TN status when the tumour is known to be ER-negative.

Figure 4 shows the predicted *BRCA1 *and *BRCA2 *carrier probabilities for an individual, ignoring FH information. Compared with the risks when the ER status of the tumour is unknown, the *BRCA1 *carrier probabilities are higher at all ages when the tumour is known to be ER-negative, and are lower when the tumour is known to be ER-positive (Figure 4a). For example, for a breast cancer diagnosed at age 30, the carrier probability is estimated to be 0.05 when the ER status is unknown, 0.11 when the tumour is ER-negative and 0.01 when the tumour is ER-positive.

Knowledge of the TN status and basal marker expression further influences mutation carrier probabilities (Figure 4b to 4d). For example, for breast cancer diagnosed at age 30, the *BRCA1 *carrier probability is estimated to be 0.19 when the tumour is TN and 0.02 when the tumour is ER-negative but not TN (Figure 4b). If both basal markers CK5/6 and CK14 are expressed on a TN tumour, the probability of carrying a *BRCA1 *mutation is 0.64 (Figure 4c). If neither CK is expressed on a TN tumour when both have been tested for, however, the carrier probability is 0.04. Carrier probabilities for the scenario where a test is available only for CK5/6 are also shown (Figure 4c). Carrier probabilities for CK14-positive and CK14-negative tumours when a test is available only for CK14 were almost identical to those obtained when a test was available only for CK5/6 (data not shown). The TN mutation status also influences *BRCA2 *mutation carrier probabilities (Figure 4d).

We also estimated carrier probabilities considering both tumour markers and FH. Table 3 and Additional file 2 (Supplementary tables S1 and S2) show mutation carrier probabilities in a simple family with an affected mother and an affected (proband) daughter. Knowledge of the ER status of either tumour substantially influences the mutation carrier probabilities. Notably, the later the age at breast cancer diagnosis, the greater the relative change in the *BRCA1 *mutation carrier probability. For example, if the mother is 70 years old when diagnosed with breast cancer, there is a threefold increase in mutation carrier probability when the tumour is ER-negative relative to when the ER status of the tumour is unknown. If the mother is 40 years old when diagnosed, the increase is less than twofold. In some cases, carrier mutation probabilities for *BRCA2 *are also altered when information on pathology of the tumour is available, because the *BRCA1 *and *BRCA2 *carrier probabilities are interdependent.

**Table 3:** Carrier mutation probabilities when the ER status of the mother is unknown, negative or positive

		Mother's age at diagnosis									
		
Proband age at diagnosis	Mother's ER status	30 years		40 years		50 years		60 years		70 years	
		
		*BRCA 1*	*BRCA2*	*BRCA1*	*BRCA2*	*BRCA1*	*BRCA2*	*BRCA1*	*BRCA2*	*BRCA1*	*BRCA2*
30 years	Unknown	*0.45*	*0.16*	*0.24*	*0.14*	*0.15*	*0.10*	*0.09*	*0.09*	*0.06*	*0.10*
	ER-negative	0.63	0.11	0.46	0.10	0.36	0.08	**0.25**	0.08	**0.19**	0.09
	ER-positive	0.13	0.25	0.08	0.17	0.04	0.11	0.04	0.10	0.03	0.10
40 years	Unknown	*0.23*	*0.14*	*0.11*	*0.10*	*0.07*	*0.07*	*0.04*	*0.06*	*0.03*	*0.07*
	ER-negative	0.38	0.11	0.24	0.08	**0.18**	0.06	**0.12**	0.05	0.09	0.06
	ER-positive	0.05	0.16	0.03	0.10	0.02	0.07	0.02	0.06	0.01	0.07
50 years	Unknown	*0.12*	*0.08*	*0.06*	*0.06*	*0.04*	*0.04*	*0.02*	*0.04*	*0.02*	*0.04*
	ER-negative	**0.22**	0.08	**0.14**	0.05	**0.11**	0.04	0.07	0.03	0.06	0.04
	ER-positive	0.02	0.09	0.01	0.06	0.01	0.04	0.01	0.04	0.01	0.04
60 years	Unknown	*0.06*	*0.07*	*0.03*	*0.04*	*0.02*	*0.03*	*0.01*	*0.03*	*0.01*	*0.04*
	ER-negative	**0.11**	0.06	0.07	0.04	0.06	0.03	0.04	0.03	0.03	0.04
	ER-positive	0.01	0.07	0.01	0.05	0.00	0.03	0.00	0.03	0.00	0.04
70 years	Unknown	*0.03*	*0.07*	*0.01*	*0.05*	*0.01*	*0.03*	*0.01*	*0.03*	*0.00*	*0.04*
	ER-negative	0.06	0.07	0.04	0.05	0.03	0.03	0.02	0.03	0.02	0.04
	ER-positive	0.01	0.07	0.00	0.05	0.00	0.03	0.00	0.03	0.00	0.04

Both mother and proband are affected and the age of diagnosis of cancer in each case is the same as the age of last follow-up. Figures in italics represent genotype-specific risk when the oestrogen (ER) status is unknown. Bold figures indicate genotype-specific risk <10% when the ER status is unknown, but >10% when the ER status is known.

**Figure 5:**
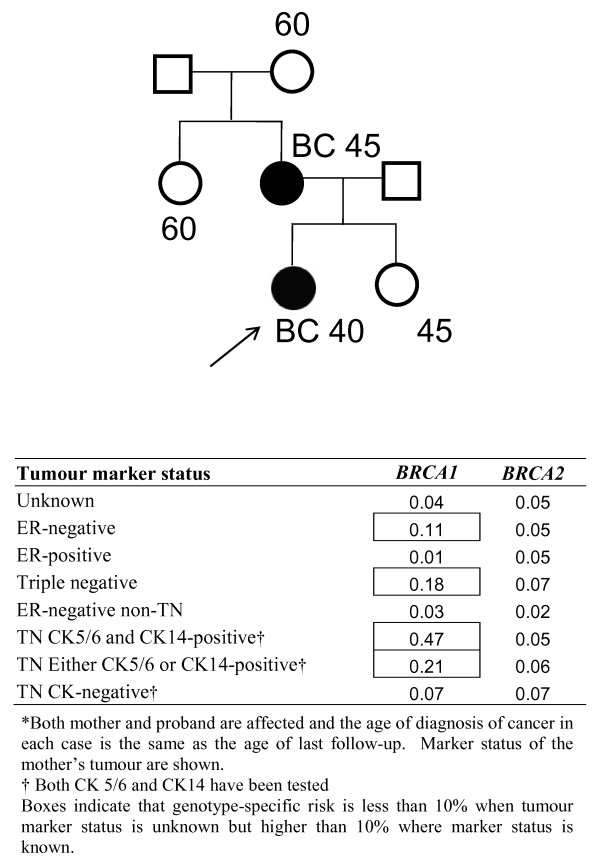
**Mutation carrier probabilities for a pedigree where sequentially more tumour pathology information is available**. CK, cytokeratin; ER, oestrogen receptor; TN, triple negative.

Figure 5 shows mutation carrier probabilities for a hypothetical pedigree in which sequentially more pathological information is available for a family member. Mutation carrier probabilities are influenced by additional information. For example, if the tumour is TN, the probability of carrying a *BRCA1 *mutation is increased. If the tumour is ER-negative but not TN, however, the probability of carrying a *BRCA1 *mutation is even less relative to when the tumour is ER-negative but of unknown TN status.

**Figure 6:**
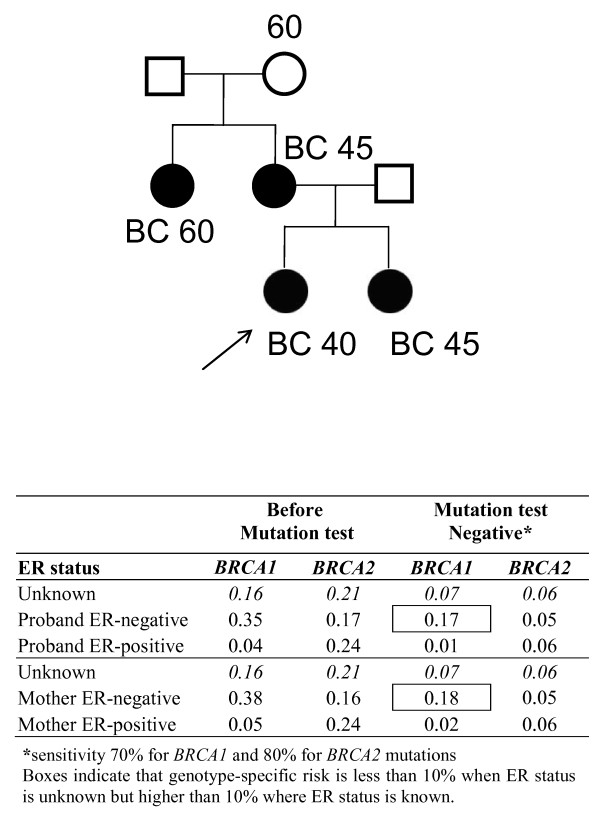
**Influence of oestrogen receptor status on carrier probabilities when the proband tested negative for mutations**. ER, oestrogen receptor.

Mutation screening for *BRCA1 *and *BRCA2 *is not 100% sensitive and can vary depending on the mutation screening methods used. BOADICEA takes into account the reduced sensitivity of mutation testing (assumed to be 70% for *BRCA1 *and 80% for *BRCA2 *for the purposes of the example [23]). Figure 6 shows a pedigree in which the proband had been tested for mutations in *BRCA1 *or *BRCA2*. As expected, the residual probability of carrying a *BRCA1 *mutation is markedly higher if the tumour in either the proband or the mother is known to be ER-negative.

## Discussion

We extended the BOADICEA model of genetic susceptibility to breast cancer and ovarian cancer to incorporate breast tumour pathology information - in particular, ER status. We divided breast cancer into distinct disease end points and used data on the proportion of ER-negative and ER-positive tumours in *BRCA1 *and *BRCA2 *carriers and the general population to derive age-specific incidences of ER-negative and ER-positive disease. Information on the TN status and expression of basal markers on TN tumours was also included in the model. Incorporating information on tumour pathology influences the predicted probabilities of carrying a *BRCA1 *or *BRCA2 *mutation, in particular *BRCA1*, and may therefore have implications for genetic testing and clinical decision-making. The methodology presented here can in principle be applied to incorporate information in risk models for other diseases where the disease can be divided into distinct phenotypes such as different tumour sites in colorectal cancer [41,42] or subphenotypes of Crohn's disease [43].

Data on the distribution of ER status for *BRCA1 *or *BRCA2 *breast cancer tumours were obtained from a study conducted by the BCLC [10]. The ER status was measured by immunohistochemistry in a single research laboratory. A potential concern is that this assay may not be representative of typical assessment of ER status conducted in routine practice. The overall proportion of ER-positive tumours in the control series of the BCLC study, albeit relatively small, was however similar to that in the SEER series, suggesting there is little relative bias in the prevalence of ER-positivity. In addition, the cases in the BCLC study were predominantly drawn from multiple breast cancer families and this might have influenced the ER status, although there is no evidence to support an association between ER status and FH [44]. The number of mutation carriers was also relatively small (182 *BRCA1 *mutation carriers and 64 *BRCA2 *mutation carriers). The age-specific proportions of ER prevalence are therefore somewhat imprecise. In the final analyses we based the *BRCA2 *estimates on the proportion of ER-positive and ER-negative tumours in the general population data, due to lack of precision in the age-specific estimates in the BCLC data. Data from larger studies, such the Consortium of Investigators of Modifiers of *BRCA1*/2 [45], may provide more precise estimates and improve the modelling of BRCA2 in the future.

To derive ER disease-specific incidences for nonmutation carriers we used the general population data from the SEER database combined with the BCLC data on mutation carriers. Data on ER status in SEER were obtained from medical records. The ER status was recorded as either positive or negative without reference to how these definitions were derived. These data may, however, be more representative of clinical situations where laboratories differ in the sensitivity with which they measure ER expression and cut-off points and scoring systems for ER positivity vary. As age-specific data were used, variation in ER expression according to age at diagnosis in control tumours is captured in our model. The observed proportion of ER-negative tumours in BCLC data among unselected controls under age 70 were 33.5%. The corresponding expected number of ER-negative tumours in the SEER database would be 35.9%. Data on TN status and cytokeratin expression in the general population were not available in these larger datasets and were derived from unselected controls from the BCLC.

We derived genotype-specific incidences for developing different pathological subtypes of breast cancer using estimates of the genotypic relative risks, mutation frequencies in the general population, estimates of the polygenic distribution and the distributions of tumour pathology markers. For this purpose, we used estimates from published studies and registry data. Uncertainty surrounds each of these parameters, however, and a degree of measurement error may also be associated with the determination of pathological markers. Although in principle it may be possible to calculate confidence intervals for the derived disease-specific incidences or carrier probabilities, this would be complex due to the unknown correlation structure between the parameters entering the model. The derived incidences are therefore based on the best currently available estimates. The cumulative risks presented in Figure 3 do not take into account competing risks of dying from nonbreast cancer causes. These risks may therefore be somewhat higher than a woman would face in reality as they assume survival to the relevant age.

Our results show that incorporating tumour marker information into BOADICEA may result in the better discrimination between *BRCA1 *carriers, *BRCA2 *carriers and nonmutation carriers. As expected, individuals with ER-negative tumours, or with ER-negative tumours diagnosed in their family members, are predicted to have a higher *BRCA1 *mutation carrier probability. The current National Institute for Clinical Excellence guidelines recommend that an affected family member is screened for mutation if the predicted carrier probability for mutations in *BRCA1 *or *BRCA2 *combined is at least 20% [46]. Knowledge of the ER status may therefore affect the decision to offer testing. For example, in a pedigree with an affected daughter and mother with ages of diagnosis at 40 and 50 years, respectively, the combined mutation carrier probabilities are 0.14 when the ER status is unknown but 0.24 if the mother's tumour is ER-negative. Similarly, the presence of an ER-positive tumour in the family may result in combined *BRCA1 *and *BRCA2 *carrier probabilities <20%.

Information on the TN status and expression of basal cytokeratins can further influence mutation carrier probabilities. In our example, the *BRCA1 *mutation carrier probability is increased over fourfold when the mother's tumour is ER^-^CK14^+^CK5/6^+ ^as compared with when it is ER-negative, but information on basal CK is unavailable. For a random 30-year-old breast cancer patient, the *BRCA1 *mutation carrier probability is approximately 10 times greater for a patient with ER^-^CK14^+^CK5/6^+ ^versus ER^-^CK^- ^breast tumour. Lakhani and colleagues argued previously that the use of CK in combination with ER status may provide a more specific test for *BRCA1 *carrier status than ER alone, because ER-negative tumours are more frequently observed amongst control tumours than are basal CKs [11]. Our results further highlight the potentially important role of basal CKs in addition to ER status for risk prediction.

In the present model we assumed that the polygenic component is identical for both ER-negative and ER-positive disease and for tumours of other subtypes; that is, the polygenes confer the same relative risk by disease subtype. Studies that have evaluated the familial relative risks of breast cancer by the ER status of the proband have in general found no significant differences in the risks for ER-negative disease and ER-positive disease [47-54]. In addition, the segregation analysis of Antoniou and colleagues estimated similar polygenic variances of breast cancer risk for *BRCA1 *and *BRCA2 *mutation carriers [23]. Recent studies, however, have demonstrated that many of the common breast cancer susceptibility variants are associated more strongly with ER-positive disease [55-58]. Further, Antoniou and colleagues have shown that modifying loci associated with *BRCA2 *cancers, but not with *BRCA1 *carriers, parallel those associated with ER-positive disease in the general population [55,59]. The polygenotypes for ER-positive and ER-negative thus cannot be perfectly correlated, but the extent of the correlation is not known. This may affect risk prediction in circumstances where the tumour subtype is available for more than one individual in a family. In principle, the methods we presented can be extended to allow for different polygenic components on ER-negative and ER-positive disease or other tumour subtypes once these can be estimated.

## Conclusions

We have developed a method for incorporating tumour marker information into risk-prediction models by subdividing the overall disease into different disease end points and have implemented this method in BOADICEA to incorporate tumour ER status, TN status, and expression of basal markers. This will be implemented in the BOADICEA web interface for use in genetic counselling. The inclusion of phenotypic markers associated with *BRCA1 *status should improve risk prediction in breast cancer.

## Abbreviations

BCLC: Breast Cancer Linkage Consortium; BOADICEA: Breast and Ovarian Analysis of Disease Incidence and Carrier Estimation Algorithm; CK: cytokeratin; ER: oestrogen receptor; FH: family history; HER2: human epidermal growth factor receptor 2; SEER: Surveillance Epidemiology; and End Results; TN: triple negative.

## Competing interests

The authors declare that they have no competing interests.

## Authors' contributions

NM, DFE and ACA contributed to the conception and execution of the project. NM carried out the analysis and wrote the manuscript. SRL was lead pathologist for the BCLC study. TRR contributed to discussions. ACA and DFE supervised the analysis and participated in writing the manuscript.

## Supplementary Material

Additional file 1 **Extending BOADICEA to incorporate tumour pathology**. Details of methods for extending BOADICEA to incorporate tumour pathology [60]. Click here for file

Additional file 2 **Subtype-specific risks and carrier probabilities**. Supplementary figure S1 shows the incidence rate of ER-negative, TN disease, and ER-negative non-TN disease: incidence rate per 100,000 individuals in (a) *BRCA1 *carriers, (b) *BRCA2 *carriers and (c) noncarriers, corresponding to women before after 1950. Supplementary figure S2 shows average cumulative risk for ER-negative disease, TN disease and ER-negative non-TN disease: average cumulative risk for (A) *BRCA1 *carriers, (b) *BRCA2 *carriers and (c) noncarriers, corresponding to women born after 1950. Supplementary figure S3 shows the incidence rate of ER-negative, TN, CK-expressing and non-expressing TN disease (both CKs tested): incidence rate per 100,000 individuals in (a) *BRCA1 *carriers, (b) *BRCA2 *carriers and (c) noncarriers, corresponding to women born after 1950. Supplementary figure S4 shows the average cumulative risk for ER-negative, TN and CK-expressing and non-expressing disease (both CKs tested): average cumulative risk for (a) *BRCA1 *carriers, (b) *BRCA2 *carriers and (c) noncarriers, corresponding to women born after 1950. Supplementary figure S5 shows the incidence rate of ER-negative, TN, and CK5/6-expressing and non-expressing TN disease (only CK5/6 tested): incidence rate per 100,000 individuals in (a) *BRCA1 *carriers, (b) *BRCA2 *carriers and (c) noncarriers, corresponding to women before after 1950. Supplementary figure S6 shows the average cumulative risk for ER-negative, TN, and CK5/6-expressing and non-expressing TN disease (only CK5/6 tested): average cumulative risk for (a) *BRCA1 *carriers, (b) *BRCA2 *carriers and (c) noncarriers, corresponding to women born after 1950. Supplementary table S1 shows carrier mutation probabilities when the ER status of the proband is unknown, negative or positive. Supplementary table S2 shows carrier mutation probabilities when the ER status of the proband and the mother are unknown, negative or positive. Click here for file
