# Supplementary material for: Incorporating tumour pathology information into breast cancer risk prediction algorithms
Source: Breast Cancer Res. 2010 May 18;12(3):R28. doi: 10.1186/bcr2576 (PMC2917017; doi:10.1186/bcr2576)
Supplement: Additional file 1 — Extending BOADICEA to incorporate tumour pathology. Details of methods for extending BOADICEA to incorporate tumour pathology [60]. [file bcr2576-S1.DOC]

# Additional file 1

Let $b_{1}(t)$ be the average incidence of breast cancer in *BRCA1* mutation carriers at age *t* in BOADICEA, $P(t)$be the value of the polygenotype and *ψ_p(t)_* the probability of having poly-genotype $P(t)$. Note that under the hypergeometric polygenic model P(t) is approximated by $P(t)=\frac{R-N}{\sqrt{N/2}}\sigma(t)$where R has a binomial distribution (2N,1/2) and $P(t)$ is therefore discrete in nature [60]. Since *ψ_p(t)_* depends only on R we omit the age “*t*” in order to simplify the notation (*ψ_p_)*. Based on our model and according to standard survival analysis theory:

$$b_{1}(t)=\frac{\sum_{p} \psi_{p}f_{1,p}^{b}(t)}{\sum_{p} \psi_{p}S_{1,p}^{b}(t-1)S_{1}^{o}(t-1)}$$

$=\frac{\sum_{p} \psi_{p}(\mu_{1}(t)+\nu_{1}(t))\exp(P(t))S_{1,p}^{b}(t-1)S_{1}^{o}(t-1)}{\sum_{p} \psi_{p}S_{1,p}^{b}(t-1)S_{1}^{o}(t-1)}$ Equation (1)

where $\mu_{1}(t)$ and $\nu_{1}(t)$ are the ER-positive and ER-negative incidences in *BRCA1* mutation carriers “free” of the polygenic effect respectively, $S_{1,p}^{b}(t)$ and$S_{1}^{o}(t)$ are the probabilities of surviving breast and ovarian cancer by age t and $f_{1,p}^{b}(t)$ is the probability of developing breast cancer at age t (i.e. the density function). We note that:

$$S_{1,p}^{b}(t)=\exp\left(-\sum_{u=0}^{t} \left((\mu_{1}(u)+v_{1}(u))\exp(P(u))\right)\right)$$

The aim is to solve for $\mu_{1}(t)$ and $\nu_{1}(t)$ (two unknowns). We further impose the constraint that the ratio of ER-positive to ER-negative tumours in *BRCA1* mutation carriers should agree with their observed ratio $\xi^{1}\left(t\right)$ obtained from the BCLC data

$\xi^{1}\left(t\right)=\frac{\sum_{p} \psi_{p}\mu_{1}(t)\exp(P(t))S_{1,p}^{b}(t-1)}{\sum_{p} \psi_{p}\nu_{1}(t)\exp(P(t))S_{1,p}^{b}(t-1)}$ Equation (2)

Setting $S_{1,p}^{b}(0)=1$, equations (1) and (2) can then be solved recursively for the baseline incidences of ER-positive and ER-negative disease ($\mu_{1}(t)$ and $\nu_{1}(t)$ respectively) free of the polygenic effect. Baseline incidences for ER specific disease in *BRCA2* mutation carriers are derived in a similar fashion.

The derivation of the baseline incidences for ER-positive and ER-negative disease in non-carriers is slightly more complicated because SEER data report the age specific distribution of tumour ER status in unselected cases, that is individuals who were not screened for *BRCA1* and *BRCA2* mutations and these distributions by definition include the *BRCA1* and *BRCA2* effects on ER-positive and ER-negative disease. Therefore, to obtain ER-specific incidences in non *BRCA1* and *BRCA2* mutation carriers we constrained the overall incidence of breast cancer in the model (over ER status, and all genetic effects) to agree with the UK population incidences according to the following equation:

i(t)=

$$\frac{\sum_{j=0}^{2} \tau_{j}\sum_{p} \psi_{p}(\mu_{j}(t)\exp P(t)S_{j,p}^{b}(t-1)S_{j}^{o}(t-1)+\nu_{j}(t)\exp P(t)S_{j,p}^{b}(t-1)S_{j}^{o}(t-1))}{\sum_{j=0}^{2} \tau_{j}\sum_{p} \psi_{p}S_{j,p}^{b}(t-1)S_{j}^{o}(t-1)}$$

Equation (3)

Where $G_{j}(t)$is the log relative risk corresponding to the major genotypes *j* = 0, 1, 2 for non-carriers, *BRCA1* and *BRCA2* carriers respectively and $\tau_{j}$, the probability for the major genotypes*.* We further impose the constraint that the age specific ratio of ER-positive disease to the ratio of ER-negative disease in the general population $\xi\left(t\right)$ agrees with the observed ratios from the SEER data:

$\xi\left(t\right)=\frac{\sum_{j=0}^{2} \sum_{p} \psi_{p}\mu_{j}(t)\exp(P(t))S_{j,p}^{b}(t-1)}{\sum_{j=0}^{2} \sum_{p} \psi_{p}\nu_{j}(t)\exp(P(t))S_{j,p}^{b}(t-1)}$ Equation (4)

and therefore we can solve recursively for the baseline incidences for ER-positive and ER-negative disease $\mu_{0}$and $\nu_{0}$ respectively.
