# Supplementary material for: Incorporating tumour pathology information into breast cancer risk prediction algorithms
Source: Breast Cancer Res. 2010 May 18;12(3):R28. doi: 10.1186/bcr2576 (PMC2917017; doi:10.1186/bcr2576)
Supplement: Additional file 2 — Subtype-specific risks and carrier probabilities. Supplementary figure S1 shows the incidence rate of ER-negative, TN disease, and ER-negative non-TN disease: incidence rate per 100,000 individuals in (a) BRCA1 carriers, (b) BRCA2 carriers and (c) noncarriers, corresponding to women before after 1950. Supplementary figure S2 shows average cumulative risk for ER-negative disease, TN disease and ER-negative non-TN disease: average cumulative risk for (A) BRCA1 carriers, (b) BRCA2 carriers and (c) noncarriers, corresponding to women born after 1950. Supplementary figure S3 shows the incidence rate of ER-negative, TN, CK-expressing and non-expressing TN disease (both CKs tested): incidence rate per 100,000 individuals in (a) BRCA1 carriers, (b) BRCA2 carriers and (c) noncarriers, corresponding to women born after 1950. Supplementary figure S4 shows the average cumulative risk for ER-negative, TN and CK-expressing and non-expressing disease (both CKs tested): average cumulative risk for (a) BRCA1 carriers, (b) BRCA2 carriers and (c) noncarriers, corresponding to women born after 1950. Supplementary figure S5 shows the incidence rate of ER-negative, TN, and CK5/6-expressing and non-expressing TN disease (only CK5/6 tested): incidence rate per 100,000 individuals in (a) BRCA1 carriers, (b) BRCA2 carriers and (c) noncarriers, corresponding to women before after 1950. Supplementary figure S6 shows the average cumulative risk for ER-negative, TN, and CK5/6-expressing and non-expressing TN disease (only CK5/6 tested): average cumulative risk for (a) BRCA1 carriers, (b) BRCA2 carriers and (c) noncarriers, corresponding to women born after 1950. Supplementary table S1 shows carrier mutation probabilities when the ER status of the proband is unknown, negative or positive. Supplementary table S2 shows carrier mutation probabilities when the ER status of the proband and the mother are unknown, negative or positive. [file bcr2576-S2.DOC]

**Supplementary Table 1**. Carrier mutation probabilities when ER status of daughter (proband) is unknown, negative or positive

|  |  | Age at diagnosis: Mother | | | | | | | | | |
| --- | --- | --- | --- | --- | --- | --- | --- | --- | --- | --- | --- |
| Age at diagnosis* | ER status | 30 | | 40 | | 50 | | 60 | | 70 | |
| Proband | Proband | ***BRCA 1*** | ***BRCA2*** | ***BRCA 1*** | ***BRCA2*** | ***BRCA 1*** | ***BRCA2*** | ***BRCA 1*** | ***BRCA2*** | ***BRCA 1*** | ***BRCA2*** |
| 30 | Unknown | *0.45* | *0.16* | *0.24* | *0.14* | *0.15* | *0.10* | *0.09* | *0.09* | *0.06* | *0.10* |
|  | ER-negative | 0.64 | 0.11 | 0.42 | 0.12 | 0.28 | 0.09 | 0.18 | 0.09 | 0.13 | 0.10 |
|  | ER-positive | 0.11 | 0.25 | 0.05 | 0.17 | 0.03 | 0.11 | 0.02 | 0.10 | 0.01 | 0.10 |
| 40 | Unknown | *0.23* | *0.14* | *0.11* | *0.10* | *0.07* | *0.07* | *0.04* | *0.06* | *0.03* | *0.07* |
|  | ER-negative | 0.45 | 0.10 | 0.25 | 0.08 | 0.16 | 0.06 | 0.11 | 0.06 | 0.07 | 0.06 |
|  | ER-positive | 0.06 | 0.16 | 0.02 | 0.10 | 0.01 | 0.07 | 0.01 | 0.06 | 0.01 | 0.07 |
| 50 | Unknown | *0.12* | *0.08* | *0.06* | *0.06* | *0.04* | *0.04* | *0.02* | *0.04* | *0.02* | *0.04* |
|  | ER-negative | 0.32 | 0.07 | 0.17 | 0.05 | 0.11 | 0.04 | 0.07 | 0.04 | 0.05 | 0.04 |
|  | ER-positive | 0.02 | 0.09 | 0.01 | 0.06 | 0.01 | 0.04 | 0.00 | 0.04 | 0.00 | 0.04 |
| 60 | Unknown | *0.06* | *0.07* | *0.03* | *0.04* | *0.02* | *0.03* | *0.01* | *0.03* | *0.01* | *0.04* |
|  | ER-negative | 0.19 | 0.06 | 0.10 | 0.04 | 0.07 | 0.03 | 0.04 | 0.03 | 0.03 | 0.04 |
|  | ER-positive | 0.01 | 0.07 | 0.01 | 0.05 | 0.00 | 0.03 | 0.00 | 0.03 | 0.00 | 0.04 |
| 70 | Unknown | *0.03* | *0.07* | *0.01* | *0.05* | *0.01* | *0.03* | *0.01* | *0.03* | *0.00* | *0.04* |
|  | ER-negative | 0.13 | 0.06 | 0.07 | 0.04 | 0.05 | 0.03 | 0.03 | 0.03 | 0.02 | 0.04 |
|  | ER-positive | 0.01 | 0.07 | 0.00 | 0.05 | 0.00 | 0.03 | 0.00 | 0.03 | 0.00 | 0.04 |

*Both mother and proband are affected and the age of onset of cancer in each case is the same as the age of last follow-up.

Boxes indicate that genotype-specific risk is less than 10% when ER status is unknown but higher than 10% where ER status is known.

**Supplementary Table 2**. Carrier mutation probabilities when ER status of both mother and daughter (proband) is known

| Age at | | Age at diagnosis: Mother  Mother | | | | | | | | | |
| --- | --- | --- | --- | --- | --- | --- | --- | --- | --- | --- | --- |
| diagnosis*  Proband ERstatus | | 30 | | 40 | | 50 | | 60 | | 70 | |
|  |  | ***BRCA 1*** | ***BRCA2*** | ***BRCA 1*** | ***BRCA2*** | ***BRCA 1*** | ***BRCA2*** | ***BRCA 1*** | ***BRCA2*** | ***BRCA 1*** | ***BRCA2*** |
| 30 | Unknown | *0.45* | *0.16* | *0.24* | *0.14* | *0.15* | *0.10* | *0.09* | *0.09* | *0.06* | *0.10* |
|  | MoER-DauER- | 0.79 | 0.07 | 0.65 | 0.07 | 0.55 | 0.06 | 0.42 | 0.06 | 0.34 | 0.08 |
|  | MoER-Dau ER+ | 0.21 | 0.23 | 0.12 | 0.16 | 0.08 | 0.11 | 0.05 | 0.09 | 0.03 | 0.10 |
|  | MoER+DauER- | 0.24 | 0.23 | 0.16 | 0.17 | 0.09 | 0.11 | 0.08 | 0.10 | 0.07 | 0.11 |
|  | MoER+DauER+ | 0.02 | 0.27 | 0.01 | 0.18 | 0.01 | 0.11 | 0.01 | 0.10 | 0.01 | 0.10 |
| 40 | Unknown | *0.23* | *0.14* | *0.11* | *0.10* | *0.07* | *0.07* | *0.04* | *0.06* | *0.03* | *0.07* |
|  | MoER-DauER- | 0.64 | 0.07 | 0.47 | 0.06 | 0.38 | 0.05 | 0.28 | 0.05 | 0.22 | 0.05 |
|  | MoER-Dau ER+ | 0.11 | 0.16 | 0.06 | 0.10 | 0.04 | 0.07 | 0.03 | 0.06 | 0.02 | 0.07 |
|  | MoER+DauER- | 0.13 | 0.15 | 0.08 | 0.10 | 0.05 | 0.07 | 0.04 | 0.06 | 0.04 | 0.07 |
|  | MoER+DauER+ | 0.01 | 0.17 | 0.01 | 0.11 | 0.00 | 0.07 | 0.00 | 0.06 | 0.00 | 0.07 |
| 50 | Unknown | *0.12* | *0.08* | *0.06* | *0.06* | *0.04* | *0.04* | *0.02* | *0.04* | *0.02* | *0.04* |
|  | MoER-DauER- | 0.50 | 0.05 | 0.36 | 0.04 | 0.29 | 0.03 | 0.22 | 0.03 | 0.17 | 0.04 |
|  | MoER-Dau ER+ | 0.04 | 0.09 | 0.02 | 0.06 | 0.02 | 0.04 | 0.01 | 0.04 | 0.01 | 0.04 |
|  | MoER+DauER- | 0.08 | 0.09 | 0.05 | 0.06 | 0.03 | 0.04 | 0.03 | 0.04 | 0.02 | 0.04 |
|  | MoER+DauER+ | 0.00 | 0.09 | 0.00 | 0.06 | 0.00 | 0.04 | 0.00 | 0.04 | 0.00 | 0.04 |
| 60 | Unknown | *0.06* | *0.07* | *0.03* | *0.04* | *0.02* | *0.03* | *0.01* | *0.03* | *0.01* | *0.04* |
|  | MoER-DauER- | 0.33 | 0.05 | 0.23 | 0.04 | 0.19 | 0.03 | 0.14 | 0.03 | 0.11 | 0.03 |
|  | MoER-Dau ER+ | 0.03 | 0.07 | 0.02 | 0.05 | 0.01 | 0.03 | 0.01 | 0.03 | 0.01 | 0.04 |
|  | MoER+DauER- | 0.04 | 0.07 | 0.03 | 0.05 | 0.02 | 0.03 | 0.02 | 0.03 | 0.01 | 0.04 |
|  | MoER+DauER+ | 0.00 | 0.07 | 0.00 | 0.05 | 0.00 | 0.03 | 0.00 | 0.03 | 0.00 | 0.04 |
| 70 | Unknown | *0.03* | *0.07* | *0.01* | *0.05* | *0.01* | *0.03* | *0.01* | *0.03* | *0.00* | *0.04* |
|  | MoER-DauER- | 0.23 | 0.05 | 0.16 | 0.04 | 0.14 | 0.03 | 0.10 | 0.03 | 0.08 | 0.04 |
|  | MoER-Dau ER+ | 0.01 | 0.07 | 0.01 | 0.05 | 0.01 | 0.04 | 0.00 | 0.03 | 0.00 | 0.04 |
|  | MoER+DauER- | 0.02 | 0.07 | 0.02 | 0.05 | 0.01 | 0.03 | 0.01 | 0.03 | 0.01 | 0.04 |
|  | MoER+DauER+ | 0.00 | 0.07 | 0.00 | 0.05 | 0.00 | 0.03 | 0.00 | 0.03 | 0.00 | 0.04 |

*Both mother (Mo) and daughter (Dau) are affected and the age of onset of cancer in each case is the same as the age of last follow-up.

Boxes indicate that genotype-specific risk is less than 10% when ER status is unknown but higher than 10% where ER status is known (- , negative or + , positive).

(A)

(B)

(C)

**Supplementary Figure 1.**

(A)

(B)

(C)

**Supplementary Figure 2.**

(A)

(B)

(C)

**Supplementary Figure 3.**

(A)

(B)

(C)

**Supplementary Figure 4.**

(A)

(B)

(C)

**Supplementary Figure 5.**

(A)

(B)

(C)

**Supplementary Figure 6.**
